# Homo- and Copolymerization of Ethylene and Norbornene with Anilido–Imine Chromium Catalysts

**DOI:** 10.3390/polym8030069

**Published:** 2016-03-01

**Authors:** Lixia Pei, Yong Tang, Haiyang Gao

**Affiliations:** 1 State Key Laboratory of Oil and Gas Reservoir Geology and Exploitation, Southwest Petroleum University, Chengdu 610550, China; tangyong2004@126.com; 2 School of Chemistry and Chemical Engineering, South China University of Technology, Guangzhou 510641, China; peilixia@scut.edu.cn; 3 Key Laboratory for Polymeric Composite & Fuctional Materials of Ministry of Education, Guangdong Provincial Key Laboratory for High Performance, Polymer-based Composites Design and Synthesis of New Polymer Materials and Application Laboratory, School of Materials Science and Engineering, Sun Yat-Sen University, Guangzhou 510275, China

**Keywords:** anilido–imine chromium, ethylene, norbornene, copolymerization

## Abstract

A series of anilido–imine chromium complexes have been used as precursors to catalyze homo- and copolymerization of ethylene and norbornene. The chromium complexes activated with methylalumoxane (MAO) exhibit good activities for homopolymerization of ethylene (E) to produce linear polyethylene and moderate activities for norbornene (N) polymerization to afford vinyl-type polynorbornene. Ethylene–norbornene copolymers with high incorporation of norbornene can be also produced by these catalysts. ^13^C NMR and differential scanning calorimetry (DSC) analyses show that the copolymers are random products, and –NNN– and –EEE– units can be observed in the microstructure of the copolymers.

## 1. Introduction

Cyclic olefin copolymers (COCs) are of great interest owing to their remarkable properties, such as high vapor and thermal resistance, excellent optical transparency, and high refractive indexes, especially poly(ethylene-*co*-norbornene)s [1,2,3,4]. In fact, these properties can be precisely controlled by varying monomer composition, sequence distribution, and the stereoregularity of norbornene units in the copolymers, which depends on the different structure of the employed catalyst including metal center and ligand structure. Compared with alternating and block poly(ethylene-*co*-norbornene)s, random copolymer is amorphous and shows excellent optical transparency and high refractive indexes, thereby can be used as optical materials. Driven by industrial application, developing catalysts for preparation of random copolymerization of ethylene with norbornene is highly desirable [1,2,3,4].

Ethylene–norbornene (E–N) copolymers were first obtained with metallocene–methylaluminoxane (MAO) based catalysts [5,6,7], and then metallocene, half sandwich or constrained-geometry Group IV catalysts (CGCs) were widely reported [8,9,10,11,12,13]. With these catalysts, random E–N copolymers with up to 70 mol % norbornene content have been described. Many non-metallocenes titanium complexes such as bis(pyrrolide-imine) titanium [14,15], bis(imino-indolide)titanium [16], bis(β-enaminoketonato) titanium [17], [2-(2,6-dialkylphenylamino)-1-phenylethoxy TiCl_2_] [18], titanium complexes bearing tridentate [O^−^NX^R^] (X = S, O, Se, P) ligands [19,20], and bis(β-diketiminato) titanium [21] were also used to effectively copolymerize ethylene (E) and norbornene (N) in the presence of MAO or modified MAO (MMAO). However, most of the non-metallocenes titanium catalysts produced alternating E–N copolymer [22], and fewer successful examples have appeared for the efficient synthesis of random copolymers with high norbornene contents (>50 mol %) [23].

Late transition metal nickel and palladium catalysts are highly active for norbornene homopolymerization, whereas they usually afford low-molecular-weight polymer for norbornene polymerization in the presence of ethylene because ethylene is a chain transfer agent. Rare catalytic systems can afford alternating or block E–N copolymers [24,25,26,27]. For examples, neutral nickel complexes containing bidentate [P,O] chelating ligands were efficient catalysts for the alternating copolymerization of norbornene with ethylene [24]. Amine-imine nickel catalyst can afford well-defined E–N block copolymers using a living polymerization technique [25]. α-Diimine palladium catalysts catalyze ethylene–norbornene copolymerization to produce alternating E–N copolymers [26,27].

Despite the widespread use of chromium catalysts for the polymerization of ethylene, only two systems for ethylene–norbornene copolymerization have been described [27,28,29]. [CpRCrMeCl]_2_ activated with MAO was reported to be capable of catalyzing copolymerization of ethylene and norbornene [28,29]. Cr(IV) alkyl complex Cr(CH_2_SiMe_3_)_4_/MAO was highly active for ethylene/norbornene copolymerizations and gave high molecular weight copolymers with –NNN– sequences [30]. Therefore, design of novel chromium catalysts for copolymerization of norbornene and ethylene is still desirable, and chromium catalysts are expected to afford E–N copolymers with different microstructures.

Herein, we reported homo- and copolymerizations of norbornene and ethylene using anilido–imino chromium complexes **1–3** (ArN = CHC_6_H_4_NAr)CrCl_2_(THF)_2_ (**1**, Ar = phenyl; **2**, Ar = 2,6-dimethylphenyl; **3**, 2,6-diisopropylphenyl) activated with MAO. The anilido–imino ligand has recently gained popularity in the field of organometallics and catalytic reactions [31,32,33,34,35,36,37,38,39]. The influences of ethylene pressure and polymerization temperature on polymerization activity and incorporation of norbornene were investigated in detail. Random E–N copolymers with successive norbornene sequences –(N)_n_– (*n* > 3) were prepared in good catalytic activity.

## 2. Experimental Section

All manipulations involving air- and moisture sensitive compounds were carried out under an atmosphere of dried and purified nitrogen with standard vacuum-line, Schlenk, or glovebox techniques.

### 2.1. Materials

Solvents were purified using standard procedures. Methylene chloride, and tetrahydrofuran (THF) were distilled from calcium hydride, and hexane was distilled from P_2_O_5_ under nitrogen. *n*-Butyllithium (*n*-BuLi) solution in hexane (2.86 M) were purchased from Aldrich (Milwaukee, WI, USA). Methylaluminoxane (MAO) solution (10 wt % in toluene) was purchased from Acros (Geel, Belgium). Norbornene (bicyclo[2.2.1] hept-2-ene; Acros) was purified by distillation over potassium and used as a solution in toluene. Polymerization grade ethylene was further purified by passage through columns of molecular sieves. Other commercially available reagents were purchased and used without purification.

Synthesis of anilido–imine chromium complexes. Anilido–imine chromium complexes were prepared according to reported method [39]. Under nitrogen, ligands were dissolved in 40mL THF in a flame-dried Schlenk flask, and *n*-butyllithium solution (2.6 M) was injected in a −78 °C dry ice/acetone bath, which was warmed room temperature overnight. CrCl_3_(THF)_3_ was added, and then stirred for 24 h at room temperature. The solvents were removed under vacuum, and the residue was extracted with CH_2_Cl_2_ (20 mL) and filtered. The filtrate was concentrated to 5 mL and mixed with hexane (50 mL). The chromium complex was cooled in a freezer several days to obtain brown crystals. 

### 2.2. Measurement

^13^C NMR spectra of polymers were carried out on a Bruker 500 MHz (Bruker BioSpin, Fällanden, Switzerland) at 120 °C *o*-C_6_D_4_Cl_2_ solution using solvent as a reference. DSC analyses were conducted with a Perkin Elmer DCS-7 system (Perkin Elmer, Waltham, MA, USA). The DSC curves were recorded at second heating curves at a heating rate of 10 °C/min and a cooling rate of 10 °C/min. Gel permeation chromatography (GPC) analyses of the molecular weight and molecular weight distribution (*PDI* = *M*_w_/*M*_n_) of the polymers at 150 °C were performed on a high-temperature chromatography, PL-GPC 220 instrument (Polymer Laboratories, Reading, Berkshire, UK) equipped with a differential refractive index (RI) detector. Wide-angle X-ray diffraction (WAXD) curve of the polymer powder was obtained using a D/Max-IIIA powder X-ray diffractometer (Parr Instrument Company, Moline, IL, USA).

### 2.3. Norbornene Polymerization

In a typical procedure, the appropriate MAO was introduced into a 50 mL round-bottom glass flask placed in an oil bath at a prescribed temperature, and then the desired amount of norbornene and toluene was added via syringe. The polymerization was initiated by injecting a chromium complex solution and the reaction mixture was continuously stirred for an appropriate period at the polymerization temperature. Polymerizations were terminated by the addition of acidic ethanol (ethanol–HCl, 95:5). The resulting precipitated polymers were collected and treated by filtering, washing with ethanol several times, and drying under vacuum at 60 °C to a constant weight.

### 2.4. Atmosphere Pressure Polymerization of Ethylene and Copolymerization

A 100 mL round-bottom glass flask was charged with toluene, prescribed amount of MAO and norbornene (for copolymerization ethylene and norbornene) at initialization temperature. The system was maintained by continuously stirring for 5 min, and then the chromium complex solution was charged into the flask. The pressure was maintained by continuously feeding ethylene gas and the reaction was carried out for a certain time. Polymerization was terminated by the addition of acidic ethanol (ethanol–HCl, 95:5). The resulting precipitated polymers were collected and treated by filtering, washing with ethanol several times, and drying under vacuum at 60 °C to a constant weight.

### 2.5. High Pressure Polymerization of Ethylene and Copolymerization

A mechanically stirred 100 mL Parr reactor (Parr Instrument Company) was heated to 150 °C for 2 h under vacuum and then cooled to room temperature. The autoclave was then charged with solution of MAO under ethylene at initialization temperature. The system was maintained by continuously stirring for 5 min, and then 2 mL solution of chromium complex in CH_2_Cl_2_ was charged into the autoclave. The ethylene pressure was raised to the specified value, and the reaction was carried out for a certain time. Polymerization was terminated by addition of acidic methanol after releasing ethylene pressure. The resulting precipitated polymers were collected and treated by filtering, washing with methanol several times, and drying under vacuum at 40 °C to a constant weight.

## 3. Results and Discussion

**Scheme 1:**
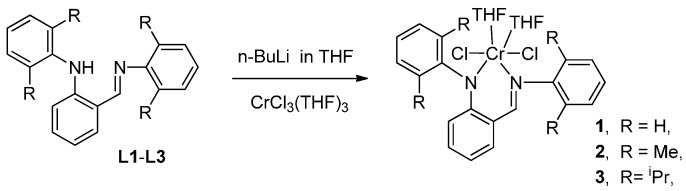
Synthesis of anilido–imino chromium complexes **1–3.**

Anilido–imino ligands were synthesized by our previously reported method [31], and anilido–imino chromium complexes **1–3** (ArN = CHC_6_H_4_NAr)CrCl_2_(THF)_2_ (**1**, Ar = phenyl; **2**, Ar = 2,6-dimethylphenyl; **3**, 2,6-diisopropylphenyl) were prepared by treating the anilido–imine ligands with *n*-butyllithium in THF and addition of CrCl_3_(THF)_3_ (Scheme 1) [39].

In the presence of MAO, anilido–imino chromium complexes **1–3** were firstly investigated as precursors for ethylene polymerization, and the polymerization data are summarized in Table 1. The steric characteristic of the chelate anilido–imino ligand plays an important role in the catalytic performances. Polymerization results in Table 1 clearly show that the order of the values of catalytic activities for ethylene polymerization is **3** > **2** > **1** under the same conditions, suggesting that bulky substituent on *N*-aryl moiety can enhance catalytic activity for ethylene polymerization. Besides, increasing the steric hindrance of the anilido–imino ligand also obviously improves polymer molecular weight.

The reaction temperature also strongly affected the catalytic activities, and the results of ethylene polymerizations using **1/**MAO at various temperatures were also summarized in Table 1. It can be seen that the catalytic activity for ethylene polymerization increased with an increase of reaction temperature gradually and reached a maximal value of 46 kg PE (mol·Cr·h)^−1^ at 40 °C (PE: polyethylene). Molecular weight decreases uniformly with the increase of polymerization temperature, which is the same as a previous observation [39].

**Figure 1:**
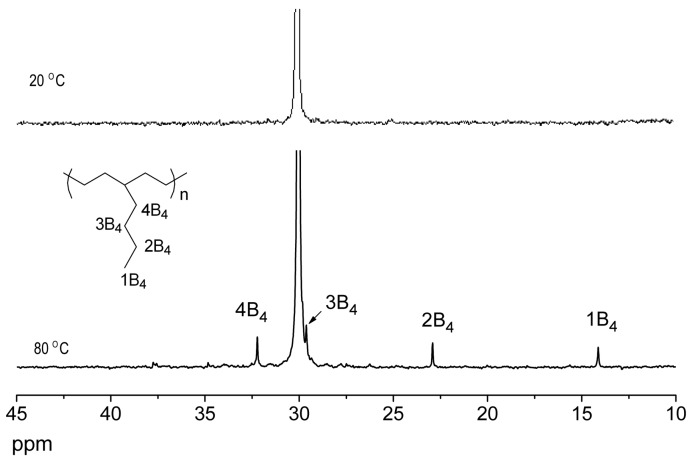
^13^C NMR spectra of PE obtained by **1**/MAO at different temperatures.

**Figure 2:**
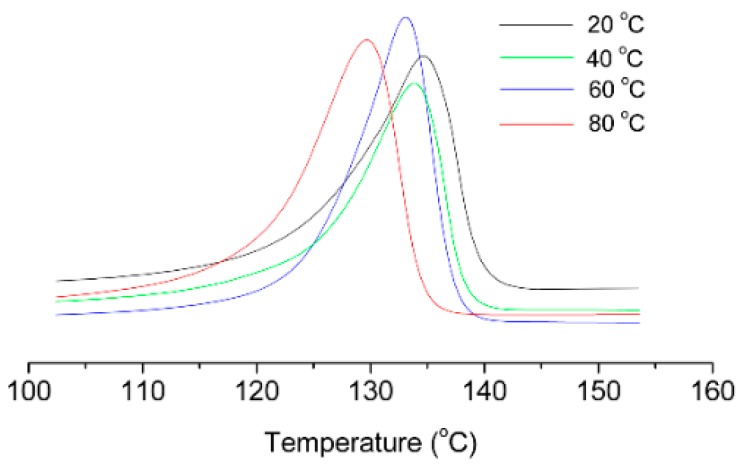
DSC curves of PE obtained by **1**/MAO at different temperatures.

^13^C NMR analysis of the PE prepared at 20 °C shows only one peak at 30.0 ppm, and no signals of branching carbons can be observed (Figure 1). At high temperature of 80 °C, a small amount of butyl branch can be observed (Figure 1). DSC analysis shows that the polymer products display a range of the melting temperatures (*T*_m_) from 129.1 to 134.7 °C (Figure 2), indicating that the produced polymers possess a linear structure.

Norboenene polymerizations were also carried out using **1–3**/MAO, and the results were listed in Table 2. In contrast to ethylene polymerization, bulky steric hindrance of the anilido–imino ligand significantly decreases catalytic activity for norbornene polymerization. Polymerization results in Table 2 clearly show that the order of the values of catalytic activities for ethylene polymerization is **1** > **2** > **3**. This can be attributed to repulsion of bulky norbornene monomer inserted into metal center and bulky ligand. Additionally, the catalytic activity for norbornene polymerization increases with an increase of reaction temperature gradually and then decreases using **1**/MAO system. The highest activity can reach 174 kg PN (mol·Cr·h)^−1^ at 60 °C. Molecular weight decreases uniformly with the increase of polymerization temperature because of an acceleration of chain transfer reaction [32].

**Figure 3:**
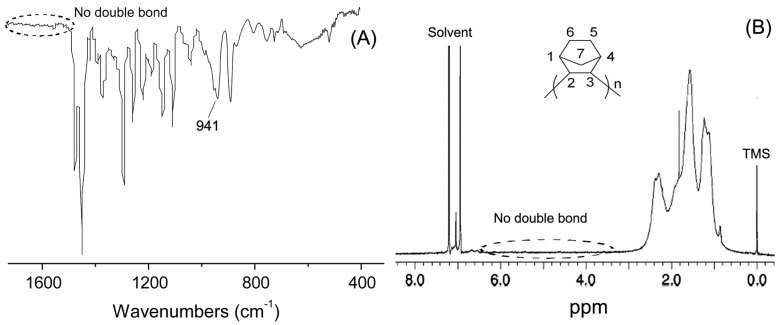
IR (**A**) and ^1^H NMR (**B**) spectroscopy of the polynorbornene (TMS: tetramethylsilane).

The obtained polynorbornenes (PNs) are white solids with relatively low molecular weight determined by high temperature gel permeation chromatography (GPC) (7.5–25.7 kg/mol). The IR spectrum revealed no traces of double bond, which often appear at 1620 ~ 1680, 966, and 735 cm^−1^, while the existence of vibration bands of bicyclics of norbornene at 941 cm^−1^ (Figure 3A). ^1^H NMR spectrum of the polynorbornene further proved no traces of any double bond (Figure 3B). Therefore, the obtained products are vinyl-addition polynorbornenes.

Good activities of anilido–imino chromium catalysts for ethylene and norbornene homopolymerizations allow us to prepare the ethylene–norbornene copolymers. Copolymerizations of ethylene and norbornene were carried out with anilido-imino chromium complexes **1–3** activated with MAO, and copolymerization data were summarized in Table 3. Considering great incorporation of ethylene in copolymer, low polymerization temperature of 20 °C was chosen to evaluate the steric effect on copolymerization of ethylene with norbornene (entries 5–7 in Table 3). Under the same conditions, bulky catalyst **3** with 2,6-diisopropylphenyl groups showed the lowest copolymerization activity, and catalyst **1** with phenyl groups afforded the highest incorporation of norbornene in copolymer. This observation can be attributed to the low activity of bulky catalyst **3** for norbornene homopolymerization.

Catalyst **1** was further selected to investigate the effects of temperature and ethylene pressure because of the high incorporation performance of norbornene. Increasing the temperature from 20 to 60 °C leads to an increase in copolymerization activity and incorporation of norbornene. In this case, the high temperature is favorable for norbornene polymerization. With an increase in ethylene pressure, the copolymerization activity and molecular weight of copolymers increase, whereas the incorporation of norbornene in copolymers decreases. Generally, the obtained E–N copolymers have high norbornene incorporation (>50 mol %), which is mostly higher than by alternating E–N copolymer obtained by non-metallocenes titanium and late transition metal (Ni, and Pd) catalysts (<50 mol %) [14,15,16,17,18,19,20,21,22,24,25,26,27].

**Figure 4:**
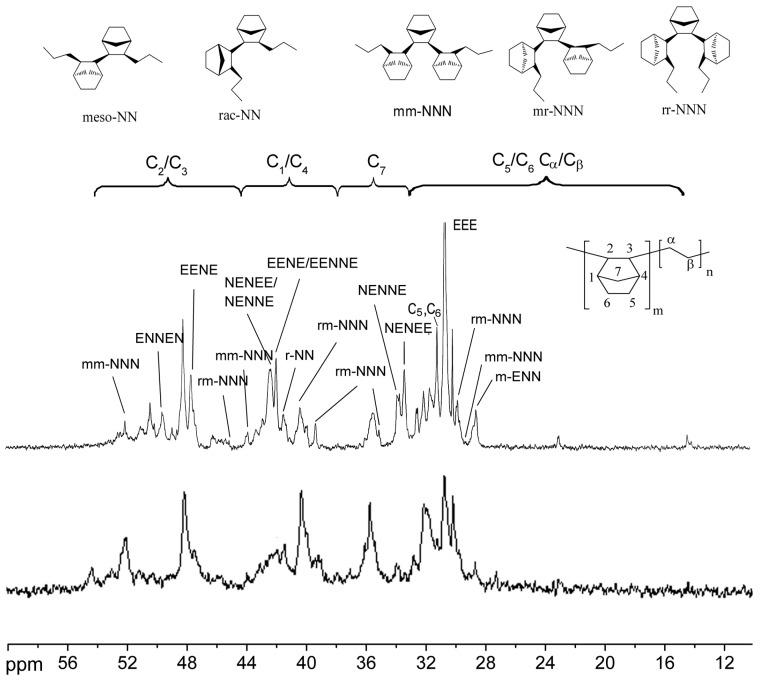
^13^C NMR spectra of E–N copolymers obtained at different ethylene pressures (up: 20 atm (entry 4), down: 0.5 atm (entry 7)).

Microstructures of the E–N copolymers were further investigated by ^13^C NMR spectroscopy. As shown in Figure 4, the spectrum of the copolymer containing norbornene incorporation of 93% obtained at 0.5 atm ethylene pressure significantly displays signals of polynorbornene, and several small peaks can be observed and assigned to isolated sequences containing ethylene units. However, the spectrum of the copolymer containing norbornene incorporation of 53% obtained at 20 atm pressure shows more complex resonances. According to previous reports and assignments [30], signal at 28.3 ppm can be assigned as characteristic of –NN– units, and –NNN– units are also presented in copolymer. Besides, –EE– units are also observed in the copolymer, and –EEE– unit are also confirmed by the characteristic peak at 29.7 ppm. The existence of –NNN– and –EEE– sequences in the microstructure of the copolymers suggests that the copolymers are random products.

**Figure 5:**
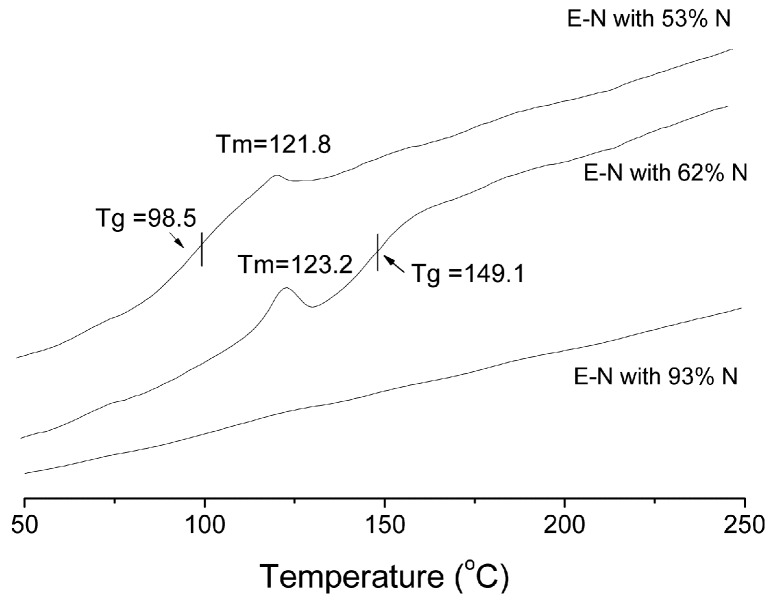
DSC curves of E–N copolymers with different norbornene incorporations.

The random structure of copolymer was also proved by the differential scanning calorimetry (DSC) analysis. As shown in Figure 5, the DSC curves of the copolymers exhibit different glass transition temperatures (*T*_g_) depending upon norbornene incorporation in the copolymers. The copolymers containing high norbornene incorporation of 93 mol % cannot show thermal transitions, which is similar to the DSC result of polynorbornene [32]. However, copolymers containing low norbornene incorporation show simultaneously *T*_g_ and melting temperature (*T*_m_). The copolymers with low norbornene incorporation show a small melt peak (~120 °C), which is a result of relative long ethylene sequences. The melting temperatures of the copolymer are lower than those of the polyethylenes, which can be attributed to long PE sequences containing very low isolated N unit ((E)_m_N(E)_n_) [7,22,23].

**Figure 6:**
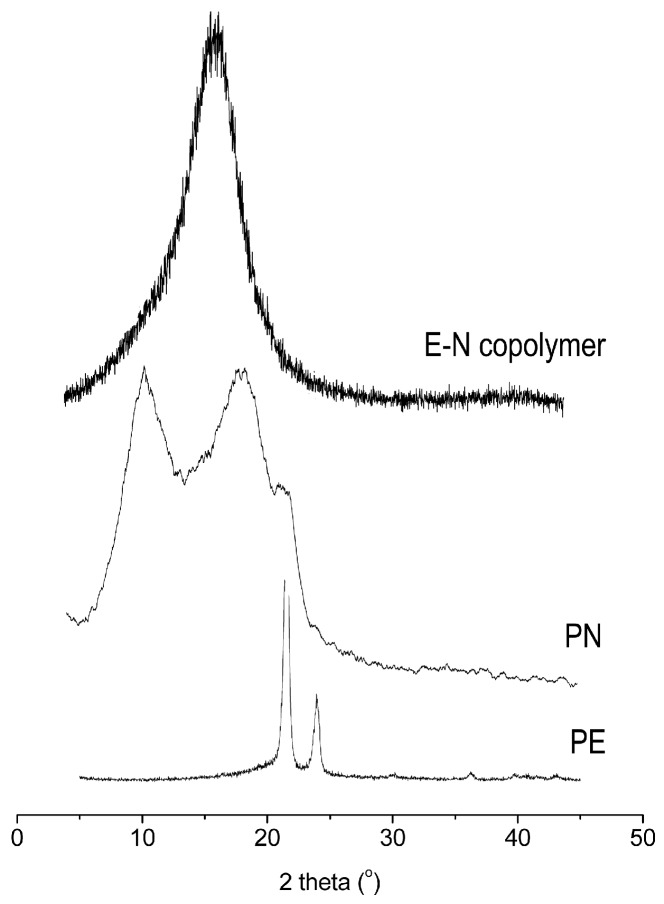
WAXD curves of polymers.

Wide-angle X-ray diffraction analysis (WAXD) (Figure 6) shows that polynorbornene is non-crystalline [32], while polyethylene is semicrystalline with two characteristic diffraction peaks at 21.3 and 23.5° [34]. However, the obtained E–N copolymer is also amorphous and has low stereoregularity because of the presence of a broad halo. This result is well consistent with ^13^C NMR and DSC analysis.

## 4. Conclusions

Anilido–imine chromium complex is a kind of highly active precursor for homo- and copolymerization of ethylene and norbornene. Anilido–imine chromium catalyst shows opposite steric effects for ethylene and norbornene polymerization. Increasing steric hindrance can enhance ethylene polymerization activity but decrease norbornene polymerization activity. The ethylene–norbornene copolymers with high incorporation of norbornene (>50 mol %) can be synthesized by changing the ethylene pressure and reaction temperature. The obtained E–N copolymers possess a random structure, and long norbornene sequences (–NNN–) as well as long ethylene sequences (–EEE–) can be observed in the microstructure of the copolymers.

## Figures and Tables

**Table 1:** Ethylene polymerizations with **1–3**/MAO.

Entry	Cat.	*T*_p_ (°C)	Yield (g)	Activity (kg PE (mol·Cr·h)^−1^)	*M*_w_^a^ (kg·mol^−1^)	*M*_w_/*M*_n_^a^	*T*_m_^b^ (°C)
1	1	20	0.34	34	762	3.54	134.7
2	1	40	0.46	46	667	2.13	133.8
3	1	60	0.37	37	444	2.46	133.1
4	1	80	0.22	22	305	2.52	129.1
5	2	40	0.68	68	845	1.95	132.9
6	3	40	1.86	186	1,020	1.88	133.6

Polymerization conditions: Cr complex, 10 μmol; Al(MAO)/Cr = 400; ethylene, 0.5 arm; reaction time, 60 min, solvent, 20 mL toluene; ^a^ Determined by high temperature gel permeation chromatography (GPC) in 1,2,4-trichlorobenzene at 150 °C; ^b^ Determined by DSC.

**Table 2:** Norbornene polymerizations with **1-3**/MAO.

Entry	Cat.	*T*_p_ (°C)	Yield (g)	Activity (kg PN (mol·Cr·h)^−1^)	*M*_w_^a^ (kg·mol^−1^)	*M*_w_/*M*_n_^a^
1	1	20	1.12	11.2	25.7	1.56
2	1	40	1.70	170	17.3	1.78
3	1	60	1.74	174	13.6	1.94
4	1	80	1.14	114	7.5	1.70
5	2	60	1.21	121	10.6	1.95
6	3	60	0.75	75	8.2	1.87

Polymerization conditions: Cr complex, 10 μmol; Al(MAO)/Cr = 400; norbornene, 4 g; reaction time, 60 min, solvent, 20 mL toluene; ^a^ Determined by high temperature gel permeation chromatography (GPC) in 1,2,4-trichlorobenzene at 150 °C.

**Table 3:** Copolymerizations of ethylene and norbornene with **1–3**/MAO.

Entry	Cat.	*T*_p_ (°C)	*P*_E_ (atm)	Yield (g)	Activity ^a^	*M*_w_^b^ (kg mol^−1^)	*M*_w_/*M*_n_^b^	Incorp._N_^c^ mol %
1	1	20	0.5	0.95	95	32.5	2.14	87
2	2	20	0.5	0.74	74	18.4	2.32	84
3	3	20	0.5	0.40	40	15.3	2.47	81
4	1	60	0.5	1.18	118	16.2	2.56	93
5	1	20	10	1.89	189	37.1	2.94	62
6	1	40	10	3.05	305	37.6	2.85	71
7	1	60	10	4.08	408	38.9	3.08	78
8	1	60	20	4.65	465	59.4	3.21	53

Polymerization conditions: Cr complex, 10 μmol; Al(MAO)/Cr = 400; norbornene, 4 g; reaction time, 60 min, solvent, 30 mL toluene; ^a^ In unit of (kg P(E-N) (mol·Cr·h)^−1^); ^b^ Determined by high temperature gel permeation chromatography (GPC) in 1,2,4-trichlorobenzene at 150 °C; ^c^ Determined by ^13^C NMR.
